# Serofibrinous pleural effusion revealing a large hydatid cyst of the liver

**DOI:** 10.11604/pamj.2022.42.198.36111

**Published:** 2022-07-12

**Authors:** Ahmed EL Mouloua, Leila Kartout

**Affiliations:** 1 Department of Pediatric Surgery, Sidi Mohamed Ben Abdellah Hospital, Essaouira, Morocco

**Keywords:** Pleural effusion, pediatric, liver hydatid cyst

## Image in medicine

Hydatid cyst of the liver remains a serious public health problem in Morocco. It is frequently endemic due to poor environmental sanitation. This benign affection can sometimes cause fatal complications, such as the intrathoracic rupture of a cyst. This is a case of a huge liver hydatid cyst, revealed by serofibrous pleurisy, which is an extremely rare condition. A 9-year-old boy, with no past medical history, was presented to the emergency department for respiratory distress, cough, and right chest pain. The pain was insidious, onset, and progressive. There was no history of fever, hemoptysis or vomica. Physical examination revealed diminished respiratory sounds, with dullness to percussion at the right lung, along-with tenderness in the right hypochondrium area. Chest X-ray film confirmed the presence of right-sided pleural effusion. Thoracoabdominal computed tomography (CT) scan was performed, and it shows a large hydatid cyst of segment VIII of the liver with a huge pleural effusion with no lung lesion. A thoracentesis revealed a yellow fluid. The hydatid cyst was treated by laparotomy and partial excision of the cyst, the diaphragmatic fistula was closed, and the pleural effusion was treated by pleural drainage. After 2 weeks, a chest X-ray showed an encysted pleural effusion, which was later treated by thoracotomy and lung decorticating.

**Figure 1:**
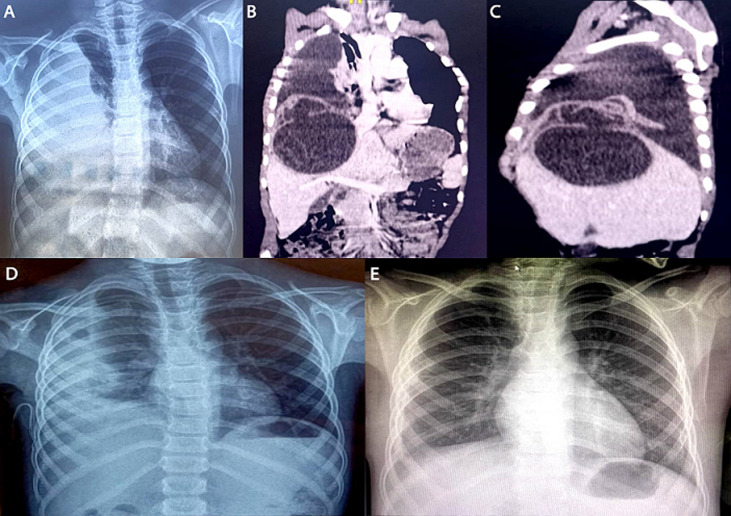
A) chest X-ray film on admission showing right-sided huge pleural effusion with no mass effect; B) coronal computed tomography scan showing huge liver hydatid cyst and a huge pleural effusion; C) sagittal computed tomography scan showing a large liver hydatid cyst communicating with the pleural cavity through diaphragmatic hole; D) chest X-ray film 2 weeks post-operative showing an encysted pleural effusion; E) chest X-ray film 3 months later showing disappearance of the pleural fluid

